# DNA Sequence Analysis of *SLC26A5,* Encoding Prestin, in a Patient-Control Cohort: Identification of Fourteen Novel DNA Sequence Variations

**DOI:** 10.1371/journal.pone.0005762

**Published:** 2009-06-02

**Authors:** Jacob S. Minor, Hsiao-Yuan Tang, Fred A. Pereira, Raye Lynn Alford

**Affiliations:** 1 Bobby R. Alford Department of Otolaryngology – Head and Neck Surgery, Baylor College of Medicine, Houston, Texas, United States of America; 2 Huffington Center on Aging and Department of Molecular and Cellular Biology, Baylor College of Medicine, Houston, Texas, United States of America; Ohio State University Medical Center, United States of America

## Abstract

**Background:**

Prestin, encoded by the gene *SLC26A5*, is a transmembrane protein of the cochlear outer hair cell (OHC). Prestin is required for the somatic electromotile activity of OHCs, which is absent in OHCs and causes severe hearing impairment in mice lacking prestin. In humans, the role of sequence variations in *SLC26A5* in hearing loss is less clear. Although prestin is expected to be required for functional human OHCs, the clinical significance of reported putative mutant alleles in humans is uncertain.

**Methodology/Principal Findings:**

To explore the hypothesis that *SLC26A5* may act as a modifier gene, affecting the severity of hearing loss caused by an independent etiology, a patient-control cohort was screened for DNA sequence variations in *SLC26A5* using sequencing and allele specific methods. Patients in this study carried known pathogenic or controversial sequence variations in *GJB2*, encoding Connexin 26, or confirmed or suspected sequence variations in *SLC26A5*; controls included four ethnic populations. Twenty-three different DNA sequence variations in *SLC26A5*, 14 of which are novel, were observed: 4 novel sequence variations were found exclusively among patients; 7 novel sequence variations were found exclusively among controls; and, 12 sequence variations, 3 of which are novel, were found in both patients and controls. Twenty-one of the 23 DNA sequence variations were located in non-coding regions of *SLC26A5*. Two coding sequence variations, both novel, were observed only in patients and predict a silent change, p.S434S, and an amino acid substitution, p.I663V. *In silico* analysis of the p.I663V amino acid variation suggested this variant might be benign. Using Fisher's exact test, no statistically significant difference was observed between patients and controls in the frequency of the identified DNA sequence variations. Haplotype analysis using HaploView 4.0 software revealed the same predominant haplotype in patients and controls and derived haplotype blocks in the patient-control cohort similar to those generated from the International HapMap Project.

**Conclusions/Significance:**

Although these data fail to support a hypothesis that *SLC26A5* acts as a modifier gene of *GJB2*-related hearing loss, the sample size is small and investigation of a larger population might be more informative. The 14 novel DNA sequence variations in *SLC26A5* reported here will serve as useful research tools for future studies of prestin.

## Introduction

The human ear is capable of perceiving sounds from 20–20,000 Hz and can distinguish a difference of as little as 0.2–0.5% [1], [2]. Such sensitivity and precision, seen also among other mammals, derives from a complex interaction of cochlear hair cells and the tectorial membrane (TM) within the organ of Corti. Stereocilia, which sit atop the outer hair cells (OHCs), embed within the TM and are deflected with the passage of sound pressure waves, triggering mechanotransducer ion channels to open and close, allowing the cell to depolarize and hyperpolarize, respectively. These changes in OHC membrane potential are converted into mechanical forces, causing the cell to rapidly lengthen and shorten, and in turn, move the basilar membrane, to result in a hundred-fold amplification of sound and a vastly improved ability to discriminate frequencies. An essential protein responsible for the electromechanical conversion in the OHC is prestin [3], [4].

The *SLC26A5* gene encodes prestin. Most genes of the SLC26 family encode anion transporters, however, mammalian prestin has only been demonstrated to be an incomplete anion transporter [4]–[6]. Further, prestin is more highly conserved among mammalian species than any other protein in its family: demonstrating 95% amino acid identity between mouse and human, compared to an average of 86% for the group [7]. Prestin also appears to have a role beyond the OHC since *SLC26A5* transcripts are found in heart, spleen, brain, and testis [8].

Homozygous prestin knockout mice display a 40–60 dB hearing loss [9]. Mutational analyses by several groups have identified many residues that are essential for and/or modulate prestin function [10]–[13]. However, linkage studies of hearing impaired families have failed so far to map *SLC26A5* as a locus associated with human hearing loss [14].

Only three sequence variations presumed to affect the coding sequence of *SLC26A5* in humans have been reported to date. In 2003, Liu et al. reported a DNA sequence variation, IVS2-2A>G, in a population that included two deaf probands homozygous for the variant. The IVS2-2A>G variant was predicted to disrupt the exon 3 splice acceptor site [15]. However, in 2005, we reported a carrier frequency among Caucasian controls of 4.1% for this variant, precluding its involvement in hereditary hearing loss [16]. In addition, the Celera database includes a missense variation, p.I67V, however, nothing is known about the hearing status of the individual carrying this variant, prohibiting audiometric assessment of its potential pathogenicity [17]. Recently, in 2007, Toth et al. reported a p.R150Q missense variation in one hearing impaired patient and his normal hearing father, suggesting the p.R150Q variant is not sufficient to cause hearing loss [18].

At the time this study was undertaken, the p.I67V variant was the only reported amino acid substitution in prestin [17] and only 447 non-coding SNPs were reported in the 49.24 kilobase genomic region of human *SLC26A5* [19]. Due to the paucity of known sequence variations in the human *SLC26A5* gene, evaluation of a patient-control cohort was undertaken to seek additional sequence variations in *SLC26A5* and explore the hypothesis that variations in prestin might modify the degree of hearing loss caused by an independent etiology. Fourteen novel DNA sequence variations in *SLC26A5* were identified, however, no evidence supporting a hypothesis that *SLC26A5* might act as a modifier gene was obtained.

## Results

### Analysis of the SLC26A5 DNA sequence

Genomic DNA isolated from peripheral blood lymphocytes or cultured lymphoblastoid cell lines of a patient-control cohort was evaluated for 11 previously reported DNA sequence variations in *SLC26A5* using DNA sequencing and allele specific methods. In the course of evaluating these 11 previously known sequence variations, 14 novel DNA sequence variations were observed. Table 1 shows the genomic DNA position, nucleotide sequence variation, polymorphism identification number, and location in the *SLC26A5* gene for all 25 *SLC26A5* sequence variations evaluated.

**Table 1:** DNA sequence variations in *SLC26A5*.

DNA sequence variation	SNP ID	Location
g.24586A>G (IVS2-2A>G)		IVS2
g.33167T>C	rs7779997	IVS5
**g.33190T>G**	rs72655379	IVS5
g.34821C>T	rs56305143*	IVS6
g.53884C>T	rs62482417	IVS10
**g.55275A>G**	rs72655388	IVS11
**g.56167A>G (p.S434S)**	rs72655380	Exon 12
**g.56381C>A**	rs72655389	IVS12
g.56388G>C	rs4604353	IVS12
**g.56417G>A**	rs72655390	IVS12
g.56471G>A	rs4285410	IVS12
**g.57105G>A**	rs72655391	IVS12
g.57132C>T	rs56373660	IVS12
g.57137T>C	rs62482415	IVS12
**g.57164G>T**	rs72655392	IVS12
**g.57365A>G**	rs72655381	IVS13
**g.66012C>T**	rs72655395	IVS14
g.67439T>C	rs12705120	IVS16
g.69036T>C	rs10273883	IVS18
**g.69743A>G (p.I663V)**	rs72655382	Exon 19
**g.69904T>C**	rs72655393	IVS19
**g.69917_69919delTCT**	rs66928926	IVS19
g.70029G>A	rs62482412	IVS19
**g.70078_70082delATATA**	rs72655394	IVS19
**g.70118A>G**	rs72655378	IVS19

Entrez database (http://www.ncbi.nlm.nih.gov) reference sequence genomic DNA positions and nucleotide (splice site/protein) variations, dbSNP SNP ID numbers [19], and genetic locations within *SLC26A5* are shown. *formerly hCG1811409, Celera database [17]. IVS = intervening sequence (intron). Novel variants are shown in bold type.

### SLC26A5 DNA sequence variations observed among patients

Sixteen different DNA sequence variations, 7 of which are novel, were identified in the *SLC26A5* gene in hearing impaired patients. Sequence variations included 15 single nucleotide substitutions, 6 of which are novel, and 1 novel small (tri-nucleotide) deletion. Fourteen of the DNA sequence variations identified among patients, 13 single nucleotide substitutions and the 1 small deletion, were found in introns, while 2 single nucleotide substitutions were found in the coding region of *SLC26A5* including g.56167A>G which predicts a synonymous change p.S434S and g.69743A>G which predicts the amino acid substitution p.I663V (Table 1, Table 2).

**Table 2:** Reference sequence allele frequencies for variations in *SLC26A5*.

DNA sequence variation	Patients	Controls					*P* value
	(n = 56)	African American (n = 52)	Asian (n = 52)	Caucasian (n = 56)	Hispanic (n = 52)	Combined (n = 212)	
g.24586A>G (IVS2-2A>G)	0.93	1.00	1.00	0.95	0.98	0.98	0.06
g.33167T>C	0.70	0.27	0.63	0.73	0.75	0.60	0.22
**g.33190T>G**	0.98	1.00	1.00	1.00	1.00	1.00	0.21
g.34821C>T	0.89	0.98	0.90	0.91	0.92	0.93	0.40
g.53884C>T	0.98	1.00	1.00	0.98	0.98	0.99	0.51
**g.55275A>G**	0.98	0.77	1.00	1.00	0.96	0.93	0.21
**g.56167A>G (p.S434S)**	0.98	1.00	1.00	1.00	1.00	1.00	0.21
**g.56381C>A**	1.00	0.98	1.00	1.00	1.00	1.00	1.00
g.56388G>C	0.98	0.90	1.00	1.00	1.00	0.98	1.00
**g.56417G>A**	1.00	1.00	1.00	1.00	0.98	1.00	1.00
g.56471G>A	0.00	0.00	0.00	0.00	0.00	0.00	1.00
**g.57105G>A**	1.00	0.94	1.00	1.00	1.00	0.99	1.00
g.57132C>T	0.89	0.98	0.90	0.91	0.92	0.93	0.40
g.57137T>C	0.86	0.92	0.98	0.80	0.90	0.90	0.34
**g.57164G>T**	1.00	1.00	1.00	1.00	0.98	1.00	1.00
**g.57365A>G**	0.98	1.00	1.00	1.00	1.00	1.00	0.21
**g.66012C>T**	0.98	0.88	1.00	1.00	1.00	0.97	1.00
g.67439T>C	0.00	0.00	0.00	0.00	0.00	0.00	1.00
g.69036T>C	0.02	0.15	0.00	0.00	0.00	0.04	0.69
**g.69743A>G (p.I663V)**	0.98	1.00	1.00	1.00	1.00	1.00	0.21
**g.69904T>C**	1.00	0.92	1.00	1.00	1.00	0.98	0.58
**g.69917_69919delTCT**	0.96	0.69	1.00	0.98	1.00	0.92	0.38
g.70029G>A	0.88	0.98	0.96	0.80	0.81	0.89	0.82
**g.70078_70082delATATA**	1.00	0.98	1.00	1.00	1.00	1.00	1.00
**g.70118A>G**	1.00	1.00	1.00	0.98	1.00	1.00	1.00

Allele frequencies of reference sequence nucleotides are provided for each DNA sequence variant. n = the number of chromosomes studied. *P* values are calculated by comparing patients to the combined control group. Novel variants are shown in bold type.

The patient carrying the heterozygous p.S434S variation in *SLC26A5* is also homozygous for the *SLC26A5* variants g.33167T>C (dbSNP rs7779997), g.56471G>A (dbSNP rs4285410), g.67439T>C (dbSNP rs12705120) and g.69036T>C (dbSNP rs10273883) (data not shown).

The patient carrying the heterozygous p.I663V variation in *SLC26A5* is also heterozygous for the *SLC26A5* variants g.33167T>C (dbSNP rs7779997), g.57137T>C (dbSNP rs62482415), and g.69917_69919delTCT (dbSNP rs66928926), and homozygous for the *SLC26A5* variants g.56471G>A (dbSNP rs4285410), g.67439T>C (dbSNP rs12705120) and g.69036T>C (dbSNP rs10273883) (data not shown).

DNA sequence variations were not found in patients in the coding or near coding regions of *SLC26A5* exons 4, 7, 8, 9, 17, 20, or 21.

### SLC26A5 DNA sequence variations observed among controls

Nineteen different DNA sequence variations, 10 of which are novel, were identified in the *SLC26A5* gene in controls. Sequence variations included 17 single nucleotide substitutions, 8 of which are novel, and 2 novel small deletions (1 tri-nucleotide deletion and 1 penta-nucleotide deletion). All DNA sequence variations identified among controls were found in introns (Table 1, Table 2). Due to the lack of DNA sequence variations in the coding or near coding regions of *SLC26A5* exons 4, 7, 8, 9, 17, 20, and 21 among patients, these exons were not analyzed in controls.

### Analysis of heterozygosity of sequence variations

Heterozygosity *(H)* for each of the DNA sequence variations evaluated in patients and controls is shown in Table 3. Allelic variation was observed for all but 2 of the 11 previously known sequence variations in *SLC26A5*: g.56471G>A; and, g.67439T>C (Table 2, Table 3). No subject was found to be homozygous for any coding region sequence variation in *SLC26A5.*

**Table 3:** Heterozygosity of sequence variations in *SLC26A5.*

DNA sequence variation	Patients	Controls				
	(n = 28)	African American (n = 26)	Asian (n = 26)	Caucasian (n = 28)	Hispanic (n = 26)	Combined (n = 106)
g.24586A>G (IVS2-2A>G)	0.14	0.00	0.00	0.11	0.04	0.04
g.33167T>C	0.32	0.38	0.35	0.32	0.35	0.35
**g.33190T>G**	0.04	0.00	0.00	0.00	0.00	0.00
g.34821C>T	0.14	0.04	0.12	0.18	0.15	0.12
g.53884C>T	0.04	0.00	0.00	0.04	0.04	0.02
**g.55275A>G**	0.04	0.38	0.00	0.00	0.08	0.11
**g.56167A>G (p.S434S)**	0.04	0.00	0.00	0.00	0.00	0.00
**g.56381C>A**	0.00	0.04	0.00	0.00	0.00	0.01
g.56388G>C	0.04	0.19	0.00	0.00	0.00	0.05
**g.56417G>A**	0.00	0.00	0.00	0.00	0.04	0.01
g.56471G>A	0.00	0.00	0.00	0.00	0.00	0.00
**g.57105G>A**	0.00	0.12	0.00	0.00	0.00	0.03
g.57132C>T	0.14	0.04	0.12	0.18	0.15	0.12
g.57137T>C	0.21	0.15	0.04	0.32	0.19	0.18
**g.57164G>T**	0.00	0.00	0.00	0.00	0.04	0.01
**g.57365A>G**	0.04	0.00	0.00	0.00	0.00	0.00
**g.66012C>T**	0.04	0.23	0.00	0.00	0.00	0.06
g.67439T>C	0.00	0.00	0.00	0.00	0.00	0.00
g.69036T>C	0.04	0.31	0.00	0.00	0.00	0.08
**g.69743A>G (p.I663V)**	0.04	0.00	0.00	0.00	0.00	0.00
**g.69904T>C**	0.00	0.15	0.00	0.00	0.00	0.04
**g.69917_69919delTCT**	0.07	0.38	0.00	0.04	0.00	0.10
g.70029G>A	0.18	0.04	0.08	0.32	0.23	0.17
**g.70078_70082delATATA**	0.00	0.04	0.00	0.00	0.00	0.01
**g.70118A>G**	0.00	0.00	0.00	0.04	0.00	0.01

Heterozygosity is provided for each DNA sequence variation. n = the number of individuals studied. Novel variants are shown in bold type.

### Analysis of sequence variants in IVS2-2A>G heterozygotes

Eight subjects (4 patients and 4 controls) included in this study were heterozygous for the previously reported g.24586A>G (IVS2-2A>G) nucleotide substitution in *SLC26A5* [15]. These 8 subjects were previously described [16] and were included here for further evaluation of the coding and near coding DNA sequence of *SLC26A5.* In addition to carrying the IVS2-2A>G variant, these subjects also carried additional sequence variations in *SLC26A5* as described in Table 4.

**Table 4:** Other DNA sequence variations observed in *SLC26A5* IVS2-2A>G heterozygotes.

DNA sequence variation	Patients				Controls			
g.24586A>G ( IVS2-2A>G)	Het	Het	Het	Het	Het	Het	Het	Het
g.33167T>C	Het	Hom Var	Hom Var	Het	Hom Ref	Het	Hom Var	Hom Var
g.57137T>C	Het	Het	Hom Var	Het	Hom Ref	Het	Het	Hom Var
g.70029G>A	Het	Het	Hom Var	Het	Hom Ref	Het	Het	Hom Var

Additional sequence variations observed in the patient and control samples heterozygous for the *SLC26A5* IVS2-2A>G variant are shown. All eight subjects were also homozygous for the variant alleles at g.56471G>A, g.67439T>C, and g.69036T>C (not shown). Het = heterozygous; Hom = homozygous; Ref = reference sequence allele; Var = variant allele.

### In silico analysis of amino acid sequence variations

The p.I663V amino acid sequence variation observed in this study, and the previously reported p.I67V [17] and p.R150Q [18] amino acid sequence variations were evaluated using the PolyPhen (http://genetics.bwh.harvard.edu/pph/) [20] and SIFT (http://blocks.fhcrc.org/sift/SIFT.html) [21] sequence analysis algorithms and by multiple sequence alignment of prestin orthologs using the HomoloGene application available through the National Center for Biotechnology Information (NCBI) web site (http://ww.ncbi.nlm.nih.gov).

PolyPhen analysis predicts the p.I67V and p.I663V variants would be benign and the p.R150Q variant may be possibly damaging. SIFT analysis predicts all three variants, p.I67V, p.R150Q, and p.I663V would be tolerated (data not shown). The degree of conservation of amino acids numbered p.I67, p.R150 and p.I663 in the human prestin amino acid sequence is shown in Table 5. All three amino acids are conserved in dog, cow, mouse and rat. Conservation of these amino acids in other species varies as shown (Table 5).

**Table 5:** Multiple sequence alignment for observed and previously reported [17], [18] amino acid sequence variations in prestin.

*Species* (gene/protein)	Protein Reference Sequence	aa 67	aa 150	aa 663
*H. sapiens* (SLC26A5)	NP_945350.1	I	R	I
*C. lupus* (SLC26A5)	XP_540393.2	I	R	I
*B. taurus* (SLC26A5)	XP_616468.2	I	R	I
*M. musculus* (Slc26a5)	NP_109652.3	I	R	I
*R. norvegicus* (Slc26a5)	NP_110467.1	I	R	I
*G. gallus* (SLC26A5)	XP_415959.2	I	R	V
*D. rerio* (slc26a5)	NP_958881.1	I	R	V
*D. melanogaster* (Prestin)	NP_649024.1	I	T	L
*A. gambiae* (AgaP_AGAP010389)	XP_559067.1	I	N	V
*C. elegans* (sulp-7)	NP_001033571.1	I	R	I
*C. elegans* (sulp-8)	NP_505493.1	I	R	V
*C. elegans* (sulp-4)	NP_505989.1	I	K	I
*C. elegans* (sulp-5)	NP_505990.2	I	R	V
*C. elegans* (sulp-3)	NP_509424.2	I	K	V
*A. thaliana* (SULTR4;1)	NP_196859.1	C	-	L
*A. thaliana* (SULTR4;2 )	NP_187858.1	C	-	L
*O. sativa* (Os09g0240500)	NP_001062644.1	C	-	L

Amino acid alignments of prestin orthologs from various species are shown using *H. sapiens* as the anchor sequence. Amino acid (aa) positions 67, 150, and 663 indicated in the top row of columns 3–5 refer to the amino acid positions in the human prestin amino acid sequence. Reference sequences used are derived from the NCBI protein database and aligned using the NCBI HomoloGene multiple sequence alignment tool (http://www.ncbi.nlm.nih.gov).

### Haplotype analysis of SLC26A5

To determine whether specific *SLC26A5* alleles might be associated with hearing loss, and therefore overrepresented in the patient group, haplotypes and haplotype blocks within *SLC26A5* were derived using HaploView 4.0 software (http://www.broad.mit.edu/mpg/haploview) [22]. Haplotypes and haplotype blocks were derived for the patient group, the control group and the combined patient-control cohort. Haplotype blocks derived for the patient-control cohort were compared to haplotype blocks derived for the International HapMap Project database markers (http://www.hapmap.org) [23].

**Figure 1:**
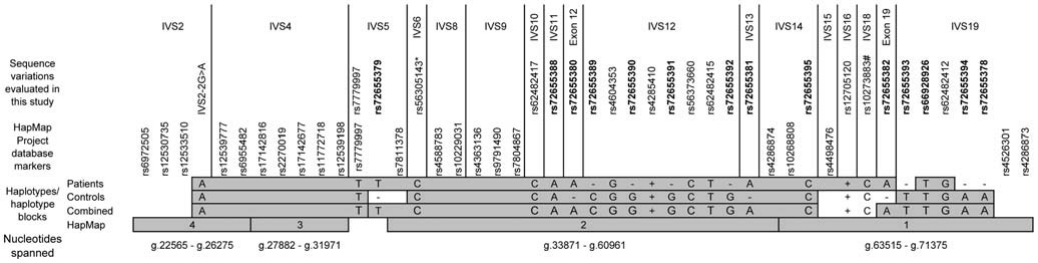
Haplotype analysis of patients and controls compared to International HapMap Project database markers. Haplotype blocks as determined by HaploView 4.0 are shown as shaded bars and compared for patients, controls, patients and controls combined (Combined), and the International HapMap Project database markers (HapMap). The location of markers within the *SLC26A5* gene is shown at the top of the figure. DNA sequence variations are shown above the haplotype blocks. Note: The only marker observed in this study that is included in the International HapMap Project database is rs7779997 in IVS5. The genomic *SLC26A5* nucleotides spanned by the haplotype blocks derived from the International HapMap Project database markers are shown at the bottom of the figure. Abbreviations and symbols used: IVS, intervening sequence (intron); *formerly hCG1811409, Celera database [17]; #, C allele noted is variant allele, not reference sequence allele; −, Variant allele not detected; +, Reference allele not detected; 1, 2, 3, 4, represent haplotype block designations derived from and assigned by HaploView 4.0 software analysis of the International HapMap Project database markers. The reference sequence (undeleted) allele of variant rs66928926 (g.69917_69919delTCT) is designated as “T” and of variant rs72655394 (g.70078_70082delATATA) is designated as “A.” Novel variants are shown in bold type.

The predominant *SLC26A5* haplotype observed in patients was also the predominant haplotype observed among controls. Although some differences are observed in the boundaries of haplotype blocks, the overall haplotype block pattern for *SLC26A5* appears similar for each of the four groups analyzed (Figure 1).

## Discussion

Twenty-three different DNA sequence variations in *SLC26A5*, 14 of which are novel, were observed. Allelic variation for two previously reported DNA sequence variations in *SLC26A5* was not observed in this study (Table 1, Table 2, Table 3). Twelve of the novel sequence variations observed are single nucleotide substitutions (SNPs) while 2 are small deletions. Four novel DNA sequence variations were found exclusively among patients. Seven novel DNA sequence variations were found exclusively among controls. Three novel DNA sequence variations, plus an additional 9 previously reported sequence variations, were found in both patients and controls.

Twenty-one of the 23 DNA sequence variations identified in this study were found in non-coding regions of *SLC26A5.* Two DNA sequence variations, both of which are novel, were found in coding regions of *SLC26A5*. Both coding region changes, a nucleotide substitution predicted not to alter the amino acid sequence of prestin, p.S434S, and a nucleotide substitution predicted to result in a missense variation, p.I663V, were found only in patients. No statistically significant difference was observed between patients and controls in the frequency of any DNA sequence variation identified in this study.

Prior to this report, only three other DNA sequence variations expected to affect the coding sequence of human *SLC26A5* were known: splice site variant IVS2-2A>G upstream of the ATG start codon [15]; missense variant p.I67V [17]; and, missense variant p.R150Q [18]. Although additional coding sequence variants will reasonably be expected to be found in future studies it is striking that such a small number of coding sequence variants have been described so far in this rather large gene whose longest isoform produces a protein containing 744 amino acids. This observation suggests a remarkably high degree of conservation of this SLC26 family protein in humans.

The IVS2-2A>G variant reported in 2003 by Liu et al. was found in that study in hearing impaired patients, including two probands homozygous for the variant, and in controls [15]. This sequence variation was suspected of disrupting the splicing of *SLC26A5* exon 3, which contains the prestin ATG start codon, thereby disrupting prestin protein production. However, the difference in allele frequency between patients and controls in the study was not statistically significant if the consanguineous proband was excluded from the analysis, p = 0.056 [15]. In an effort to further analyze the IVS2-2A>G transition, we reported in 2005 a patient-control cohort that demonstrated a carrier frequency for the IVS2-2A>G transition among deaf Caucasians of 5.6% and a carrier frequency among control Caucasians of 4.1%, p = 0.66 [16]. In addition, we identified five possible alternate splice sites in intron 2 which could potentially compensate for the IVS2-2A>G variation [16]. If the IVS2-2A>G variant was in fact associated with hearing loss in humans, based on the carrier rate of 4.1% observed among Caucasians, the contribution to hereditary hearing loss in humans of this *SLC26A5* variant could reasonably be expected to be greater than that of mutations in *GJB2*, encoding Connexin 26, which have a carrier rate among Caucasians of approximately 3% [24]. Based on these observations, it is curious that *SLC26A5* has not been mapped by linkage studies as a DFN locus [14]. Further investigation is needed into what role, if any, the IVS2-2A>G variation in *SLC26A5* might have in human hearing loss [16].

The p.I67V amino acid substitution was reported in the Celera database [17]. Because nothing is known about the health or hearing status of the individual(s) carrying this variant, its potential pathogenicity is unknown. The PolyPhen protein analysis tool predicts the p.I67V variation to be benign and the SIFT protein analysis tool predicts the p.I67V variation to be tolerated (data not shown). Multiple sequence alignment demonstrates a high degree of conservation of this amino acid (Table 5). In this study, the p.I67V variant was not observed among patients and was not sought among controls. Further investigation of the p.I67V amino acid variant is needed to assess what impact, if any, it might have on prestin production, trafficking and/or function.

The previously reported p.R150Q amino acid substitution was observed in a hearing impaired patient and his normal-hearing father, suggesting that this amino acid substitution is not sufficient to cause hearing loss [18]. PolyPhen analysis suggests the p.R150Q variant may be possibly damaging, however, SIFT analysis predicts this variation would be tolerated (data not shown). Multiple sequence alignment demonstrates a fairly high degree of conservation of this amino acid (Table 5). In this study, the p.R150Q variant was not observed among patients and was not sought among controls. Further analysis of the p.R150Q amino acid variant is needed to assess what role, if any, it might have in hearing loss.

The novel DNA sequence variation g.56167A>G which is predicted to represent a synonymous p.S434S variation was found in this study in a patient with bilateral, progressive, mild to moderate sensorineural hearing loss who is also homozygous for the controversial p.V37I variation in *GJB2* [25]. This patient also demonstrates mildly prominent vestibular aqueducts bilaterally, a finding not consistent with a *GJB2*-based etiology and suggestive of a more complex etiology for the hearing loss. Because this sequence variation does not result in an amino acid substitution, it would not be predicted to be pathogenic unless it disrupts splicing of the *SLC26A5* transcript. Since the p.S434S variation does not predict an amino acid substitution, analysis by PolyPhen and SIFT is not warranted. Further analysis of the g.56167A>G DNA sequence variant is required to assess what impact, if any, it might have on prestin production, trafficking and/or function.

The novel DNA sequence variation g. 69743A>G which is predicted to result in a p.I663V amino acid substitution was observed in this study in a patient with bilateral, profound sensorineural hearing loss who is also homozygous for the pathogenic c.35delG mutation in *GJB2*. The detection of a homozygous c.35delG mutation in *GJB2* in this patient provides a clear etiology for this patient's hearing loss without the need to invoke additional contributing factors. Further, PolyPhen analysis predicts the p.I663V variant would be benign and SIFT analysis predicts p.I663V would be tolerated by the prestin protein (data not shown). Multiple sequence alignment demonstrates some degree of conservation of this amino acid, especially among mammals, however, in some other species the isoleucine at this position is replaced by a valine (Table 5). Further analysis of the p.I663V amino acid variant is needed to assess what impact, if any, this variant might have on prestin production, trafficking and/or function.

Haplotype analysis of *SLC26A5* was conducted to determine whether novel or unusual haplotypes or haplotype blocks occurred in the patient population that might suggest an association of particular *SLC26A5* alleles with hearing loss. Haplotype analysis using HaploView 4.0 software [22] revealed haplotypes in patients similar to those in controls and haplotype blocks in the patient-control cohort similar to those generated from International HapMap Project data [23]. Although slight differences in the boundaries of the haplotype blocks are observed between the patient, control, combined patient-control cohort, and International HapMap Project database marker groups, the overall haplotype block pattern is similar between the four groups. As such, these data fail to detect any major haplotypes or haplotype block structures unique to patients. It is likely that the slight differences in haplotype blocks observed are related to the small population size utilized in this study. Evaluation of additional patient and control populations in future studies may reveal haplotype blocks more similar to those derived using markers from the International HapMap Project database [23].

In summary, 12 novel SNPs and 2 novel small deletions are reported in the human *SLC26A5* gene encoding prestin. Although no significant difference in the frequency of any DNA sequence variation was found between patients and controls and no data was found to support the hypothesis that *SLC26A5* may act as a modifier of *GJB2-*based hearing loss in this population of hearing impaired patients, the population included in this study was small. Future studies on larger patient and control populations may yield more informative results. Identification of the 14 novel DNA sequence variations described here should facilitate future studies of *SLC26A5* and the role of the encoded prestin protein in human hearing sensitivity and hearing loss.

## Materials and Methods

### Ethics Statement

This work was approved by the Baylor College of Medicine Institutional Review Board (IRB). Written informed consent was obtained from all subjects, as directed by the IRB.

### Subjects

Patients and controls were identified as previously described and reported [16], [25]. Of the 28 patients included in this study: 22 carry DNA sequence variations in *GJB2*, encoding Connexin 26, of known, controversial, or uncertain pathogenicity [25]; 4 carry the previously reported *SLC26A5* IVS2-2A>G variant [16]; and, 2 were suspected of carrying another non-coding sequence variation in intron 2 of *SLC26A5* (data not shown). The ethnicity of patients is not always known. The 22 patients carrying known or potential mutations in *GJB2* were included in this study specifically to test the hypothesis that *SLC26A5* might act as a modifier gene of *GJB2-*based hearing loss.

Controls were obtained from the Baylor Polymorphism Resource (http://www.bcm.edu/blg/showned.cfm?01-106). The control population includes 4 different ethnic groups. Ethnicity of individuals in the control population was self-identified. Four controls previously reported to carry the *SLC26A5* IVS2-2A>G variant [16] were included in this study specifically to search for additional *SLC26A5* variants in these individuals.

### Reference sequences

Entrez nucleotide database (http://www.ncbi.nlm.nih.gov/) sequence NT_079596.2 was used as the reference sequence for *SLC26A5*. Numbering of nucleotides in *SLC26A5* begins with position 1 of the Entrez sequence as position g.1.

Entrez protein database (http://www.ncbi.nlm.nih.gov/) sequence NP_945350.1 was used as the reference sequence for prestin. Numbering of amino acids in prestin begins with position 1 of the Entrez sequence as position p.1.

### DNA sequence variation nomenclature

DNA and protein sequence variations are named according to standard nomenclature recommendations [26], [27].

### Specimen collection

As previously described [16], [25], blood samples were collected from all subjects by peripheral venipuncture in lavender top (EDTA) and/or yellow top (ACD) tubes. For some patients and all control specimens, lymphoblastoid cell lines were established from blood samples collected in yellow top (ACD) tubes by standard Epstein Barr virus mediated transformation.

### DNA isolation

As previously described [16], [25], genomic DNA was isolated from blood samples collected in lavender top (EDTA) tubes using the PUREGENE® DNA Purification Kit for whole blood and bone marrow and from cultured cells using the PUREGENE® DNA Purification Kit for cells, tissue, body fluids, and Gram-negative bacteria (Gentra Systems, Inc., Minneapolis, Minnesota, USA) according to the manufacturer's specifications.

### DNA sequence analysis

#### Of patients

The coding and near coding regions of all exons in all isoforms of *SLC26A5* were sequenced in the patient population as described below in the section entitled *PCR and DNA sequencing* except that analysis of the IVS2-2A>G variant was conducted as reported previously [16]. Novel sequence variants identified in patients in this study were confirmed either by sequencing in the opposite direction or by RFLP or TaqMan® analysis as described below in the sections entitled *RFLP analysis* and *TaqMan® analysis*, except that the g.55275A>G variant was also confirmed by bidirectional sequencing. Due to the size of the study population and the *SLC26A5* gene, at least one occurrence of every previously reported variant assayed by sequencing was confirmed by a second sequencing reaction in the opposite direction or by TaqMan® analysis, however, additional occurrences were only confirmed when deemed appropriate for control purposes or necessary based on the appearance of the initial sequence electropherogram.

#### Of controls

Screening of the control population for the sequence variants observed in patients was conducted by sequencing as described below in the section entitled *PCR and DNA sequencing* or, as indicated, by RFLP or TaqMan® analysis as described below in the sections entitled *RFLP analysis* and *TaqMan® analysis*. Novel sequence variants identified only in controls in this study were confirmed either by sequencing in the opposite direction or by a second confirmatory sequencing reaction in the same direction as the first. However, due to the size of the study population and the *SLC26A5* gene, previously reported variants assayed by sequencing were only confirmed by a second sequencing reaction either in the opposite direction or in the same direction as the first when deemed necessary for confirmation based on the appearance of the initial sequence electropherogram.

#### PCR and DNA sequencing

To minimize the number of PCR reactions required to amplify the coding and near coding genomic sequence of *SLC26A5,* a combination of standard and long PCR was used. Standard PCR was conducted with Taq polymerase (GE Healthcare, Piscataway, NJ, USA). Long PCR was conducted with the Expand Long Template PCR System (Roche Applied Science, Indianapolis, IN, USA). PCR reactions were conducted as follows: initial denaturation at 94°C was followed by 40 cycles of amplification which included denaturation at 94°C, annealing at temperatures ranging from 53°C to 60°C dependent upon primer sequence, and extension at 70°C for standard PCR and 68°C for long PCR. Amplification was followed by a final extension at 70°C for standard PCR and 68°C for long PCR.

Amplified fragments were sequenced using either PCR or inset sequencing primers with fluorescently labeled dideoxynucleotides by chain terminator (Sanger) methods using the ABI BigDye Terminator v3.1 Cycle Sequencing Kit (Applied Biosystems, Foster City, CA, USA) as previously described [16]. Sequencing reactions were analyzed on an ABI Prism 3130 Genetic Analyzer according to manufacturer's specifications (Applied Biosystems, Foster City, CA, USA).

Primer sequences and detailed PCR conditions will be provided upon request.

#### RFLP analysis

Restriction digests were performed according to the restriction enzyme manufacturer's specifications. PCR fragments containing position g.33190 were digested with restriction enzyme BstNI (New England Biolabs, Ipswich, MA, USA). When the consensus sequence was present, the 350 bp PCR fragment was not digested. When the T>G variant sequence was present, the digestion resulted in two fragments of 250 bp and 100 bp.

PCR fragments containing position g.53884 were digested with restriction enzyme EcoO109I (New England Biolabs, Ipswich, MA, USA). When the consensus sequence was present, the digestion resulted in two fragments of 420 bp and 180 bp. When the C>T variant sequence was present, the 600 bp PCR fragment was not digested.

PCR fragments containing position g.66012 were digested with restriction enzyme DraIII (New England Biolabs, Ipswich, MA, USA). When the consensus sequence was present, the digestion resulted in two fragments of 225 bp and 140 bp. When the C>T variant sequence was present, the 365 bp PCR fragment was not digested.

#### TaqMan® analysis

TaqMan® assays were performed according to the manufacturer's recommendations. Premade TaqMan® SNP Genotyping Assays (Applied Biosystems, Foster City, CA, USA) were used for the detection of sequence variants g.33167T>C (Assay ID C_25989012_10), g.34821C>T (Assay ID C_25742018_10) and g.69036T>C (Assay ID C_25986987_10). Custom TaqMan® SNP Genotyping Assays were designed for analysis of sequence variants g.55275A>G and g.67439T>C.

#### In silico DNA analysis

Missense amino acid sequence variations were analyzed for potential pathogenicity using the PolyPhen (http://genetics.bwh.harvard.edu/pph/) [20] and SIFT (http://blocks.fhcrc.org/sift/SIFT.html) [21] sequence analysis algorithms. Both algorithms use information from multiple sequence alignments of similar proteins to predict whether an amino acid substitution at any given position would be tolerated. PolyPhen also incorporates analysis of structural information into predictions. Protein sequence P58743 (version P58743.1, GI:20139418) from the UniProtKB/Swiss-Prot (http://www.ebi.ac.uk/swissprot/) and NCBI GenPept databases (http://www.ncbi.nlm.nih.gov) was used as the reference sequence for these analyses.

Multiple sequence alignments for prestin orthologs from multiple species were derived using the HomoloGene application on the National Center for Biotechnology Information (NCBI) web site (http://www.ncbi.nlm.nih.gov). Species and protein reference sequences included in the multiple sequence alignment are shown in Table 5.

#### dbSNP submission

All novel DNA sequence variants identified in *SLC26A5* in this study were submitted to dbSNP (http://www.ncbi.nlm.nih.gov) and assigned rs numbers in Build 130 as indicated in Table 1.

#### Statistical analysis

Two-tailed *P* values associated with allele frequencies in patients and controls were calculated using the Fisher's exact test analysis of 2×2 contingency tables algorithm available through GraphPad QuickCalcs Online Calculator for Scientists (http://www.graphpad.com/quickcalcs/index.cfm).

#### Heterozygosity

Heterozygosity *(H)* is calculated as the number of heterozygous individuals divided by the total number of individuals tested.

#### Haplotype analysis

Haplotype analysis was conducted using HaploView 4.0 software (http://www.broad.mit.edu/mpg/haploview) [22]. International HapMap Project data markers (http://www.hapmap.org) [23] and DNA sequence variations evaluated in *SLC26A5* in this study were uploaded into HaploView 4.0 software for analysis as singletons in Linkage format. Blocks were defined using the four gamete rule. Haplotypes above 1% were examined.
